# Resolution of post-trauma conjunctival inclusion cyst using 0.5% timolol maleate eyedrops

**DOI:** 10.5935/0004-2749.2023-0313

**Published:** 2024-05-09

**Authors:** Rosalia Antunes-Foschini, Lívia Feitosa Alves

**Affiliations:** 1 Hospital das Clínicas, Faculdade de Medicina de Ribeirão Preto, Universidade de São Paulo, Ribeirão Preto, Brazil; 2 Department of Ophthalmology, Otorhinolaryngology, and Head and Neck Surgery, Faculdade de Medicina de Ribeirão Preto, Universidade de São Paulo, Ribeirão Preto, Brazil

A 70-year-old woman presented with a conjunctival cyst after a towel hit her left eye (LE) 2 weeks prior. She was initially prescribed with retinyl acetate, aminoacids, methionine, and chloramphenicol ointment as well as a lubricant eyedrop for 7 days; these did not provide resolution. Ophthalmological examination revealed a translucent conjunctival inclusion cyst surrounded by several small vessels in the LE temporal side (Figure 1A). Following administration with 0.5% timolol maleate eyedrops twice daily, the inclusion cyst completely resolved 2 weeks later. Figure 1B shows the conjunctiva of the LE 50 days after the patient’s initial presentation.

**Figure:**
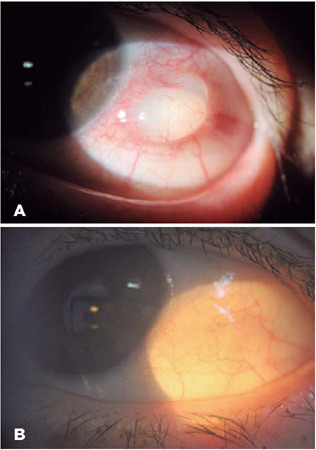

Although rare, acquired conjunctival cysts^(1)^ may develop due to local trauma. Surgical excision is usually indicated^(2)^. Before excision, timolol maleate was initially prescribed, hypothesizing that the vessels surrounding the cyst might possess beta-adrenergic receptors similar to pyogenic granulomas and capillary hemangiomas^(3,4)^, which are effectively treated with beta-blockers.
